# Epidemiological landscapes in the Amazon: exploring the association between land use, environmental indicators, and vector-borne diseases

**DOI:** 10.1590/0102-311XEN067024

**Published:** 2025-06-09

**Authors:** Ana Claudia Rorato Vitor, Cláudia Torres Codeço, Maria Isabel Sobral Escada

**Affiliations:** 1 Instituto Nacional de Pesquisas Espaciais, São José dos Campos, Brasil.; 2 Programa de Computação Científica, Fundação Oswaldo Cruz, Rio de Janeiro, Brasil.

**Keywords:** Environmental Indicators, Amazonian Ecosystem, Diseases Vectors, Indicadores Ambientales, Ecosistema Amazónico, Vectores de Enfermedades

## Abstract

This study investigates the characteristics of municipalities in the Legal Amazon that may be associated with the incidence of vector-borne diseases using environmental and land use indicators and cluster analysis. We identified and described six groups of Amazonian municipalities with similar environmental and agrarian characteristics. Based on a set of epidemiological indicators, we explore the incidence of neglected vector-borne tropical diseases in these groups. Results show a great environmental heterogeneity in the Amazonian municipalities, with well-defined profiles in the obtained groups. Moreover, they show the relation of economic activities and environmental degradation with the spread of diseases. This approach can comprehensively show the environmental and health challenges of regions, contributing to the development of more effective conservation strategies to be adapted to the specific needs of each municipality profile.

## Resumen

A partir de indicadores ambientales, este estudio evalúa técnicas de uso del suelo y análisis de conglomerados, las características de los municipios de la Amazonía Legal que pueden estar asociadas con la incidencia de enfermedades vectoriales. Para ello, identifica y describe seis grupos de municipios amazónicos con características ambientales y agrarias similares. Con base en un conjunto de indicadores epidemiológicos, se evalúa la incidencia de enfermedades tropicales vectoriales desatendidas en estos grupos. Los resultados muestran una gran heterogeneidad ambiental de los municipios amazónicos, con perfiles diferenciados y bien definidos en los grupos evaluados. Además, demuestran la relación de las actividades económicas y la degradación ambiental con la propagación de enfermedades. Este enfoque permite una visión integral de los desafíos ambientales y sanitarios que enfrenta la región y contribuye al desarrollo de estrategias de conservación más efectivas y adaptadas a las necesidades específicas de cada perfil de municipio.

## Introduction

The Amazon is recognized worldwide as one of the most biodiverse and ecologically important regions on the planet, playing a crucial role in regulating the regional and global climate ^1^ ^,^ ^2^. The remarkable cultural, linguistic, and human settlement diversity in the Brazilian Amazon encompasses several Indigenous ethnicities, quilombola, and riverine communities and a variety of urban centers, ranging from small urbanized nuclei, small and medium-sized municipalities to industrialized urban centers linked to the Brazilian Industrial Pole (such as Manaus, Amazonas State) or to the chains of exported raw materials (ore and agricultural products), such as Belém (Pará State) ^3^. However, increasing human pressure on the biome has raised significant concerns about its long-term sustainability. The region increasingly endures the impacts resulting from forms of occupation that suppress the forest, leading to increased temperature and extreme drought events such as the one experienced in 2023 ^4^ ^,^ ^5^ and increased susceptibility to fires ^6^ ^,^ ^7^ ^,^ ^8^. Studies suggest that parts of the Amazon rainforest could undergo significant structural changes in the future due to climate change and intense conversions of land use and cover, accelerating carbon emissions and impacting biodiversity, human health, and socioeconomic and cultural values of the region ^9^ ^,^ ^10^.

The current environmental situation of the Amazon stems from multiple and continuous human actions that affect the region and compromise its environmental integrity ^11^ ^,^ ^12^ ^,^ ^13^. The main factors that affect the region and lead to forest disturbance and loss include logging, the use of fire to manage areas with agricultural activities, illegal land grabbing, mineral exploration, and the expansion of the agricultural frontier and infrastructure projects on forest areas ^14^ ^,^ ^15^ ^,^ ^16^. The occurrence and intensity of these factors threaten the integrity of the biome and undergo modulation by a complex interaction between social, land, and economic factors associated with the historical occupation of Amazonian municipalities.

Over the last decades, the occupation of the Amazon has occurred in a heterogeneous way both in time and space, suffering the influence of government policies, economic interests, and socio-environmental pressures. While some areas were colonized in a planned way by implementing agricultural settlement and regional development programs by the government, others were occupied spontaneously and disorderly by migration processes of populations from the South and Northeast, especially in search of land and economic opportunities ^17^ ^,^ ^18^. Occupation policies failed to accommodate in rural settlements the entire population contingent that migrated to this region in the 1970s, whether from the Brazilian National Institute of Colonization and Agrarian Reform (INCRA, acronym in Portuguese) or from private colonization projects. This diversity in the occupation of the Amazon landscape has resulted in a variety of land use patterns, ranging from extensive deforested areas for agricultural activities to areas of traditional occupation, such as Indigenous, quilombola, and river lands, in addition to areas of integral protection and sustainable management with extractive use.

This context shows that the environmental and landscape transformations in the Amazon are closely linked to the dynamics of the regional agrarian economy that has been structured over the years ^13^. The heterogeneity of land occupation patterns and the dynamics of the agrarian economy have significant implications on environmental degradation patterns in the region. Moreover, the Amazon already suffers the impacts of global climate change, evinced by the sharp increase in temperatures, changes in rainfall patterns, and the intense droughts and fires that have recently affected the biome ^10^ ^,^ ^19^ ^,^ ^20^. Such changes result in reduced forest resilience, changes in species composition, mass tree mortality, and irreversible loss of biodiversity ^21^ ^,^ ^22^.

In turn, the modes of production associated with different agricultural systems and the transformations in the landscape and biodiversity due to them can directly impact human health ^13^ ^,^ ^23^. For example, sustainable management areas for extractive use, which keeps extensive areas of forest standing, play a crucial role in conserving biodiversity and maintaining ecosystem services. On the other hand, the conversion of forests to agricultural land can lead to habitat loss of animal species, which will seek food and shelter in areas of human occupation, increasing exposure and the risk of zoonotic diseases that can be transmitted to humans. Generally speaking, deforestation and landscape fragmentation can increase the abundance of disease reservoirs and vectors in contact with human communities ^24^ ^,^ ^25^ ^,^ ^26^. Studies project that the risk of vector-borne diseases in the Amazon suffer the influence of climate and environmental changes ^27^ ^,^ ^28^. Thus, several economic activities can impact the landscape, contributing to the emergence and re-emergence of diseases ^29^. Leishmaniasis, malaria, Chagas disease, and dengue constitute prevalent neglected tropical diseases in the Amazon that stem from infectious agents or parasites that predominantly affect vulnerable populations ^30^. These diseases indicate social and environmental vulnerability ^13^. Some studies point to the association between the dynamics of communicable diseases such as malaria, Chagas disease, dengue, and yellow fever and the loss of biodiversity and changes in the landscape ^28^ ^,^ ^31^. According to these studies, the intensification of pressure on this biome generates critical socio-environmental conditions that can trigger the emergence of new infectious diseases from wild reservoirs. Therefore, environmental and human health in the Brazilian Amazon are inextricably linked, and the regional agrarian economy plays a central role in this interconnection, affecting both the health of local populations and the health of the Amazon ecosystem as a whole. Understanding the heterogeneity of factors that can lead to major environmental transformations in the Amazon, including forest loss, is essential to promote a more sustainable development for the entire region.

Given this scenario, a detailed understanding of the factors associated with environmental changes that affect the municipalities of the Brazilian Amazon is essential to provide information that can help guide public conservation policies and support decision-making aimed at the sustainable management of natural resources and human health. Thus, this study aims to answer the following question: can Amazonian municipalities that have similar environmental and land use characteristics be associated with a specific set of vector-borne diseases? To elucidate this question, we developed a typology with categories of municipalities in the Brazilian Amazon that have environmental and agrarian characteristics in common based on a set of environmental and land use indicators. This approach can find the main economic factors involved in the transformations of landscapes and their environmental conditions for each category obtained by mainly considering the processes of forest loss and their implications in the transmission of vector-borne diseases, such as malaria, Chagas disease, leishmaniasis, and dengue. To achieve this objective this study used available data and information on a variety of environmental, land use, and epidemiological indicators, including deforestation, forest degradation, areas burned by fires, and mining ^32^.

## Methods

### Study area

The study area included all municipalities entirely within the Brazilian Legal Amazon. The Brazilian Legal Amazon comprises a political-administrative region covering about 5 million km^2^ and corresponds to approximately 58.9% of the Brazilian territory. This region comprises the states of Acre, Amapá, Amazonas, Pará, Rondônia, Roraima, Mato Grosso, Tocantins, and part of Maranhão. Regarding Brazilian biomes, Brazilian Legal Amazon houses the entire Amazon biome (composed mainly of tropical forests), 20% of the Cerrado biome (composed predominantly of savanna vegetation), and about 40% of the Pantanal biome (floodplains) in the State of Mato Grosso. The Amazon rainforest plays a central role in maintaining the regional hydrological cycle, contributing to the recycling and transport of moisture inside and outside the region ^33^. Deforestation from multiple transitions of land use and cover poses an immediate threat to forests, with deforestation for pastures being the most frequent and high-impact transition in the Brazilian Amazon ^34^ ^,^ ^35^. Other high-impact transitions, which occur less frequently or in a smaller area, but with great importance, include conversions to agriculture ^34^ and mining ^36^. In general, the intensification or decrease in the degradation of the Amazon biome responds to changes in environmental policies, such as the monitoring of deforestation and activities associated with the illegal land market, the incentive for economic activities, the construction of infrastructure that enable the flow of agricultural production (such as roads, ports, and hydroelectric plants), and the oscillation of commodity prices. According to the Satellite Monitoring of Deforestation in the Brazilian Amazon Forest (PRODES, acronym in Portuguese), the estimated value of accumulated deforestation for 2023 totaled 9,001km^2^, representing a reduction of 22.37% when compared to the deforestation rate consolidated by PRODES 2022, which equaled 11,594km^2^ for the nine states of Brazilian Legal Amazon, but still remaining at a high level. The large and rapid transformations of the forest landscape can lead to imbalances in this ecosystem and, consequently, changes the transmission cycles of vector-borne diseases due to habitat loss and the proximity of the human population to forest remnants, vectors, and reservoirs, leading to greater exposure to these diseases.

### Environmental and epidemiological indicators

A set of environmental indicators was used to characterize the environmental profile of Amazonian municipalities, observing the following dimensions: environmental changes, land use and land cover (LULC), and climatic anomalies. The dimension of environmental change includes the indicators of deforestation, forest degradation, fires, mining, and forest fragmentation. The LULC dimension is represented by indicators of agriculture, pastures, urbanized area, and secondary vegetation. Indicators of precipitation and temperature anomalies represent the dimension of climatic anomalies. The indicators were calculated for all Brazilian Legal Amazon municipalities and were constructed from open and consolidated databases. The association of clusters of municipalities with the predominance of neglected vector-borne tropical diseases was also examined. For this, a set of epidemiological indicators of incidence of cutaneous and visceral leishmaniasis, malaria, Chagas disease, and dengue for the period 2015-2019 was used, consulting Brazilian Health Informatics Department (DATASUS). The incidence rate measures the frequency of the disease in dynamic populations, and was estimated by dividing the number of cases by the population in the middle of the period and multiplying it by five (number of years of observation). All environmental and epidemiological indicators in this study are described in detail in Rorato et al. ^32^ and available for access in: https://zenodo.org/records/7098053. The environmental, land use, and epidemiological indicators are summarized in Box 1.

**Box 1:** Environmental, land use, and epidemiological indicators.

DIMENSION	INDICATOR	DESCRIPTION	UNIT	PERIOD	ID
Environmental change	Deforestation	The total area of deforestation in the municipality from 2010 to 2016 is divided by its original forest area	km^2^/km^2^	2010-2016	desmat
Environmental change	Forest degradation	The total area of forest degradation in the municipality from 2007 to 2017 is divided by its original forest area	km^2^/km^2^	2007-2017	degrad
Environmental change	Fire	Ratio of the area of the municipality that suffered at least one fire from 2012 to 2017	km^2^/km^2^	2012-2017	fogo
Environmental change	Mining	Ratio of the area of the municipality used for mining (industrial and manual) in 2017	km^2^/km^2^	2017	mine
Environmental change	Fragmentation	Ratio of the area of the municipality classified as natural vegetation nucleus in 2017	km^2^/km^2^	2017	nucleo
Environmental change	Fragmentation	Ratio between the total border (perimeter) of the patches of natural vegetation and the square root of the total area of these patchesin the municipality in 2017	m/sqrt(m^2^)	2017	borda
LULC	Secondary vegetation	Ratio of the area in the municipality covered by secondary vegetation in 2017	km^2^/km^2^	2017	veg_sec
LULC	Pasture	Ratio of the area in the municipality classified as pasture in 2017	km^2^/km^2^	2017	pasto
LULC	Agriculture	Ratio of the area in the municipality classified as agriculture in 2017	km^2^/km^2^	2017	agric
LULC	Urban area	Ratio of the area in the municipality classified as urban in 2017	km^2^/km^2^	2017	urb
Climatic anomalies	Precipitation	Average area of positive precipitation anomaly during the 2007-2017 dry seasons, divided by total area	m^2^/m^2^	2007-2017	precp+
Climatic anomalies	Precipitation	Average area of negative precipitation anomaly during the 2007-2017 dry seasons, divided by total area	m^2^/m^2^	2007-2017	precp-
Climatic anomalies	Temperature	Average area of positive temperature anomaly during the 2007-2017 dry seasons, divided by total area	m^2^/m^2^	2007-2017	temp+
Epidemiological	Vector-borne diseases	Incidence of Chagas disease, cutaneous leishmaniasis, visceral leishmaniasis, dengue, malaria	Incidence rate	2015-2019	inc_

ID: identifier; LULC: land use and land cover.

### Cluster analysis

To identify groups of municipalities in the Amazon that face similar environmental changes, a cluster analysis was conducted with all the environmental variables above. This unsupervised technique aims to divide a set of data into similar groups, commonly called clusters, to maximize intra-cluster homogeneity and inter-cluster heterogeneity ^37^. The *k-means* clustering method, a non-hierarchical technique widely employed due to its simplicity and effectiveness, in which the user determines a priori the number of clusters *k* was used. In this method, *k* objects (municipalities) are selected as initial centers, and the remaining objects are allocated based on similarity, ensuring the shortest distance between each object and the center of the cluster. The center of each cluster, known as center of gravity, is calculated as the average of the objects in the cluster. In this study, the Euclidean squared distance was used as a measure of similarity. This method grouped municipalities with similar environmental characteristics based on the values of environmental and agrarian indicators.

Before analysis, a logarithmic transformation was applied in the indicators to improve variable distribution normality. Then, the logarithmic indicators were standardized using the *z-score* method to improve the accuracy of the *k-means* algorithm, which was based on the Euclidean distance of the observations ^38^ ^,^ ^39^. The optimal number of clusters was determined using the Elbow method, which consists of plotting the explained variation as a function of the number of groups. The choice of the number of groups is made by looking at the graph and choosing the number of groups according to the Elbow of the curve, that is, the number of groups in which the plotted curve begins to resemble a straight line. All analyses and visualizations were conducted using the *stats* and *factoextra* packages on R (http://www.r-project.org) and Quantum Gis 3.0 (https://qgis.org/en/site/).

### Modeling the distribution of vector-borne diseases in environmental clusters

Generalized linear models were used to identify associations between the incidence of vector-borne diseases and the clusters of municipalities generated by the environmental classification. Models with Poisson, negative binomial, and negative binomial with zero inflation distributions were tested on R, using the *MASS* and *pscl* libraries. All had as outcome the total number of cases in the municipality (from 2015 to 2019), and as offset, the log of the estimated population in 2017; the classification of the environmental cluster was the only explanatory variable. For dengue and leishmaniasis, the negative binomial model had the best quality of fit, as measured by the Akaike information criterion. The absent convergence for malaria and Chagas disease required the zero inflation model.

## Results and discussion

### Environmental and land use classification of municipalities

**Figure 1:**
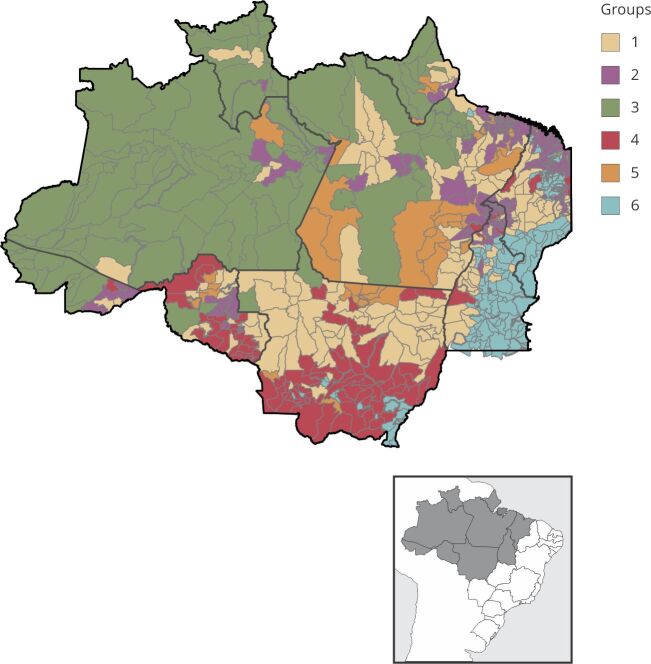
Environmental classification of municipalities in the Brazilian Legal Amazon, in 2017, obtained by cluster analysis (*k-means)*.

**Figure 2:**
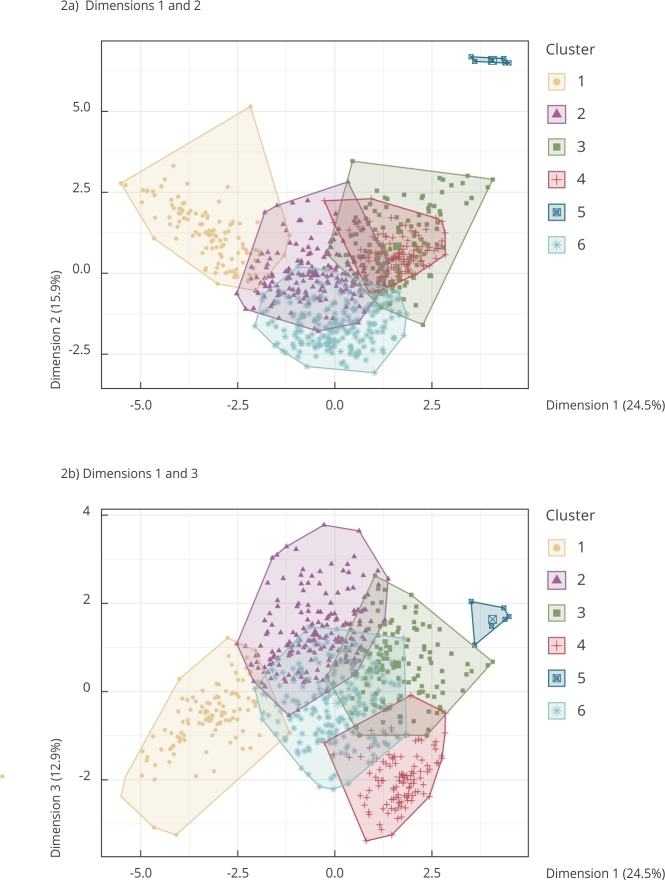
Distribution of the municipalities of the Brazilian Legal Amazon in the multivariate space generated by the environmental variables of the study.

Note: the colors indicate the groups obtained from the *k-means* cluster analysis.

**Figure 3:**
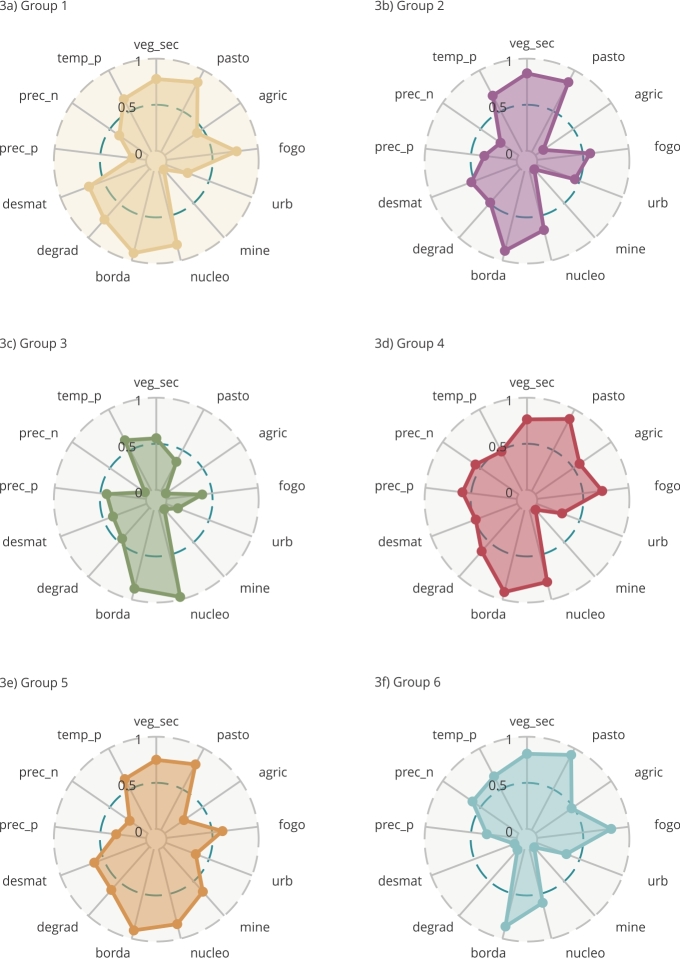
Radar graphs indicating the distribution of environmental variables in each municipality cluster. Brazilian Legal Amazon.

Note: the values indicate the mean of each indicator for each group. To improve the visualization of the results in this graph, we applied logarithmic transformation followed by scaling between 0 and 1 (using the min-max method) on the original variables.

**Figure 4:**
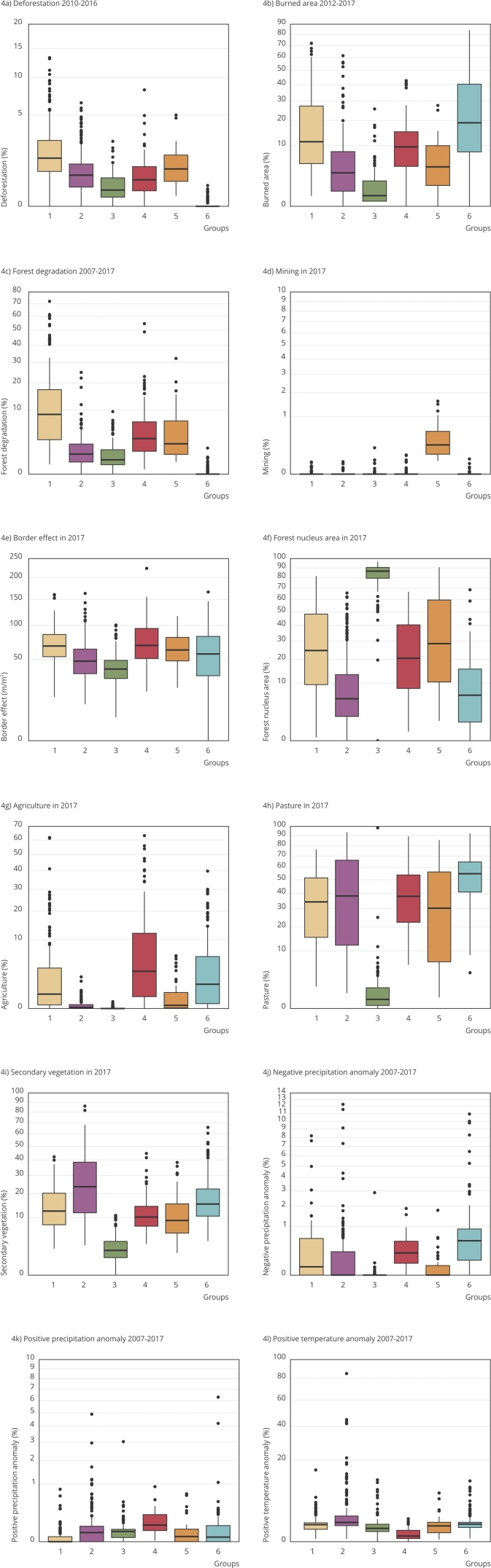
Boxplots of environmental indicators in the clusters of Brazilian Legal Amazon municipalities.

Note: the axes increase quadratically to improve the visibility of the differences. The upper and lower limits correspond to the first and third quartiles, and the line inside the box represents the median. The data beyond the end of the limits are outliers and plotted as points.

Based on the Elbow method, six groups (*k* = 6) were found in our analysis. Each group is composed of a set of municipalities with a similar environmental profile, the spatial distribution of which is shown in Figure 1. Figure 2 shows the distribution of the groups of municipalities in the multivariate space, the axes of which constitute the first three principal components. Regarding the separation of the groups, one can observe that groups 1, 3, and 6 are well separated regarding axis 1, whereas group 6 stands out regarding axis 2 (Figure 2a). Axis 2 also separates group 5 from all others. Groups 2 and 4 show better evince regarding axis 3 (Figure 2b). Our results show a great environmental heterogeneity of the Amazonian municipalities (Figures 3 and 4), and in general, with well-defined profiles in each cluster. The environmental characteristics of each group are summarized in Figures 3 and 4 and are discussed below.

Group 1 is composed of 169 municipalities and constitutes the second largest cluster. The municipalities of this group mostly lie in the states of Pará, Mato Grosso, and Maranhão and some isolated municipalities in the interior of the states of Amapá, Roraima, Amazonas, Rondônia, Tocantins, and Acre (Figure 1). This group stands out for its municipalities with the highest averages of deforestation, forest degradation, and forest edge effect between all groups. It includes municipalities such as Santarém (western Pará State), Novo Progresso and Jacareacanga (southwestern Pará State), and Colniza (northwestern Mato Grosso State), which have large forest areas, often in conservation units or Indigenous lands. However, part of these municipalities, such as Novo Progresso and Colniza, also feature among the 10 municipalities that have more deforestation in the period considered up to 2023 ^40^. The group also has the second highest average in burned areas (Figures 3 and 4). In the case of deforestation, practices such as large-scale mechanized agriculture and extensive livestock farming are driven by the land market and by the conversion of forest areas to meet the demand for commodities.

Group 2 is made up of 201 municipalities, the largest cluster of all. The municipalities of this group mainly lie in the states of Maranhão, Tocantins, Pará, Acre, and Rondônia (Figure 1). They are more numerous in the State of Pará and mainly lie in the northeast region (Bragantina zone) of this state, neighboring the municipalities of the State of Maranhão that are also included in this group. These are municipalities in which a large part of the agricultural systems are carried out on fallow systems. In these systems, soil fertility is recovered by fallow, which consists of abandoning the land and letting secondary vegetation grow after harvesting at intervals that may vary from region to region ^5^ ^,^ ^41^. This group includes riverine municipalities in the states of Amazonas and Pará, in which the fallow system is also common practice. The municipalities of this group have the highest averages of secondary vegetation area, urbanization, and positive temperature anomaly (Figures 3 and 4). These municipalities showed little deforestation in the analyzed period either because they have large areas of primary forest or have small remnants of primary forest but a large proportion of secondary vegetation replacing it.

Group 3 consists of 111 municipalities mainly lying in the states of Amazonas, Roraima, Acre, Pará, and Amapá (Figure 1). The municipalities of this group lie in the western region of the Amazon, with the highest proportion of forest area, standing out for its highest percentage averages of forest nuclei areas when compared to the other groups. On the other hand, they have the lowest values for fire, pastures, agriculture, urbanization, and negative precipitation anomaly (Figures 3 and 4), indicating lower environmental disturbances and more intact forest.

Group 4 is composed of 90 municipalities mainly in areas of consolidated agricultural use, focusing on the states of Mato Grosso and Rondônia (Figure 1). These municipalities are environmentally characterized by the highest averages of areas under forest edge effect (tying with Group 1), agriculture, and positive precipitation anomalies. In the State of Mato Grosso, the indicated municipalities may have large proportions of non-forest or seasonal forest dominant vegetation type areas. Moreover, this grouping includes the second largest area of pastures, forest degradation, and negative precipitation anomalies (Figures 3 and 4).

Group 5 is composed of 43 municipalities, mainly in the states of Pará, Rondônia, Mato Grosso, and Amapá (Figure 1). This group stands out for its municipalities with the highest average percentage of mining area and the second highest average percentage of deforestation in relation to the other groups. We also highlight the predominance of areas under edge effect and forest degradation (Figures 3 and 4). This group includes municipalities such as Itaituba and Jacareacanga (western Pará State) and São Felix do Xingu and Ourilândia do Norte (eastern Pará State), in which illegal mining activity has greatly intensified in recent years, including inside Indigenous lands such as that of the Kayapó and Munduruku, significantly impacting local populations ^42^. These municipalities have large forest areas due to the presence of conservation units and Indigenous lands, and, in deforested areas, the main coverage is pasture, although the group fails to show the highest average of pasture areas of the clusters.

Group 6, the third largest cluster, is composed of 159 municipalities, mainly in the states of Tocantins, southern Maranhão, and Mato Grosso (Figure 1). These small municipalities lie in a transition region with the Cerrado biome and may show large patches of Cerrado vegetation type. This group stands out for its highest averages of negative precipitation anomaly, pasture areas, and burned area. It also stands out with the second highest average annual agricultural area, secondary vegetation, and positive temperature anomalies (Figures 3 and 4).

### Association of vector-borne diseases with environmental classification

**Figure 5:**
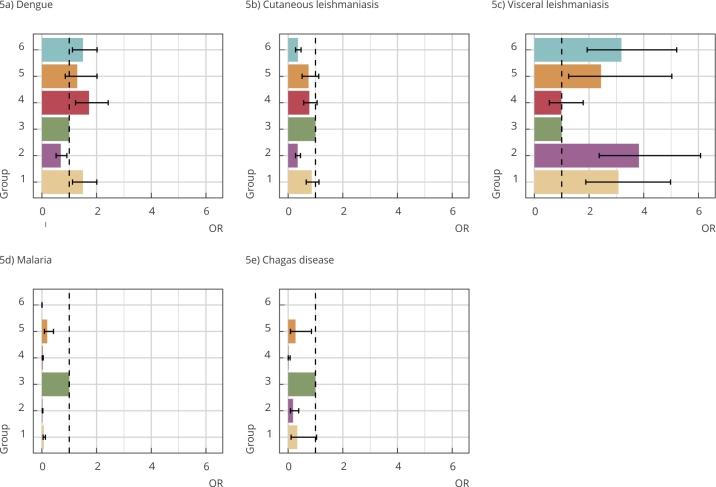
Association between the incidence of vector-borne diseases and the groupings generated by the environmental classification of municipalities in the Brazilian Legal Amazon.

At this stage, we explore the assumption that the environmental and land use classification produced by the study is related to the distribution of the incidence of some vector-borne diseases in the region. For the five diseases studied, the inclusion of the variable “group” resulted in a better fit, measured by deviance analysis. Figure 5 and Table 1 show the odds ratios of occurrence of diseases by environmental group, calculated with group 3 as a reference; this group is the set of municipalities with the highest preservation of the biome.

OR: odds ratio. Note: the bar indicates the chance ratio of occurrence of the case in the cluster compared to cluster 3 (higher ecological integrity). The description of the groups is in Figures 3 and 4 and the location, in Figure 1.

**Table 1:** Result of the adjustment of generalized linear models.

Disease/Group	OR	95%CI
Dengue		
1	1.50	1.12-2.01
2	0.69	0.52-0.91
3	1.00	1.00-1.00
4	1.72	1.23-2.42
5	1.29	0.85-2.02
6	1.50	1.12-2.02
Cutaneous leishmaniasis		
1	0.86	0.66-1.13
2	0.35	0.27-0.45
3	1.00	1.00-1.00
4	0.77	0.57-1.06
5	0.75	0.51-1.12
6	0.36	0.27-0.47
Visceral leishmaniasis		
1	3.08	1.88-4.98
2	3.82	2.37-6.08
3	1.00	1.00-1.00
4	0.98	0.55-1.79
5	2.44	1.25-5.03
6	3.19	1.93-5.21
Malaria		
1	0.07	0.04-0.13
2	0.02	0.01-0.03
3	1.00	1.00-1.00
4	0.02	0.01-0.05
5	0.20	0.09-0.42
6	0.00	0.00-0.01
Chagas disease		
1	0.33	0.11-1.04
2	0.18	0.08-0.39
3	1.00	1.00-1.00
4	0.01	0.00-0.07
5	0.27	0.09-0.85
6	0	NO

95%CI: 95% confidence interval; NO: not observed; OR: odds ratio.

**Figure 6:**
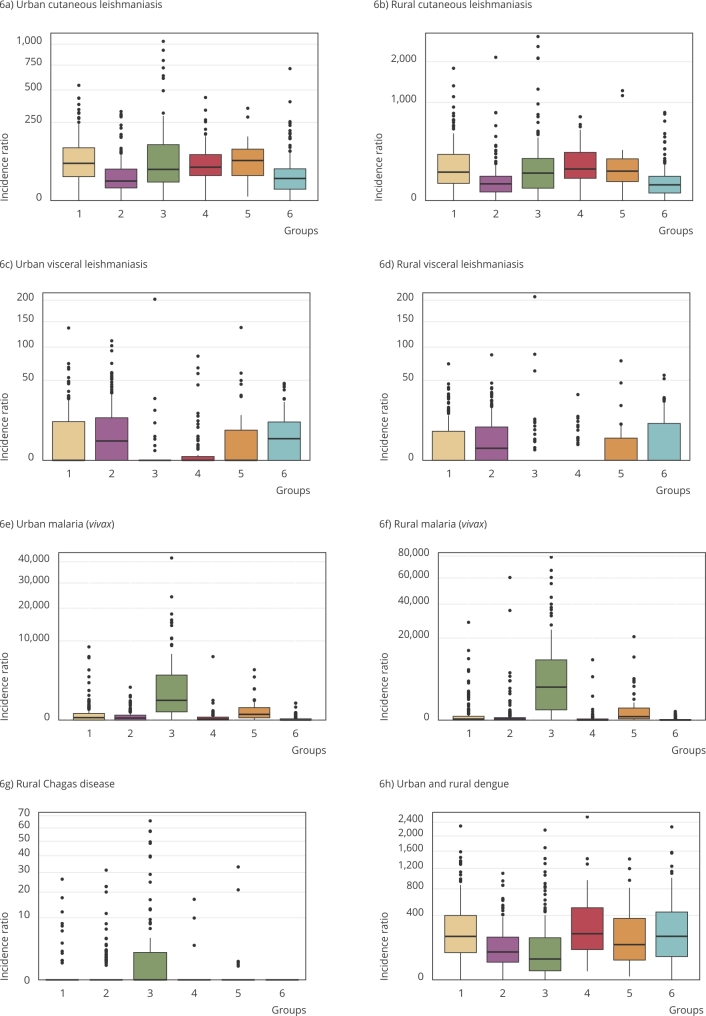
Boxplots of epidemiological indicators in the clusters of Amazonian municipalities.

Note: the axes increase quadratically to improve the visibility of the differences. The upper and lower limits correspond to the first and third quartiles, and the line inside the box represents the median. The data beyond the end of the limits are outliers and plotted as points.

Dengue and cutaneous leishmaniasis had a high incidence in all environmental groups (Figure 6). However, dengue has the highest incidences in groups 1, 4, and 6 (Figure 5a), which are concentrated in the south and southeast of the Amazon Region, areas of great environmental transformation sharing characteristics such as the high percentage of burned area in the period, forest fragmentation, strong agriculture, pasture, higher proportion of secondary vegetation, and a higher occurrence of negative precipitation anomalies between groups. On the other hand, dengue has the lowest incidence in group 2, followed by group 3. These groups share a smaller burned area, lower forest degradation and fragmentation, and a smaller extension of agricultural area.

Cutaneous leishmaniasis had a statistically similar incidence in groups 1, 3, 4, and 5, which represents 53% of all municipalities. These municipalities have a large proportion of forest remnants, with varying degrees of deforestation and forest fragmentation. Only groups 2 and 6 had a lower chance of this disease (Figure 5b). These groups share some characteristics: smaller forest nuclei areas, larger areas with secondary vegetation, and greater occurrence of positive temperature anomaly. Historically, cutaneous leishmaniasis is associated with Amazonian municipalities with a large proportion of forest area, especially in extractive communities. Nevertheless, the advance of human occupation and urbanization in the region has led to cutaneous leishmaniasis in other landscapes ^43^, which explains its wide distribution throughout the region, but still related to the presence of forest. New outbreaks of this disease are associated with areas with recent deforestation for the implementation of agriculture ^44^, which is consistent with results.

Visceral leishmaniasis had a higher incidence in the municipalities of groups 1, 2, 5, and 6 in a very different pattern from cutaneous leishmaniasis (Figure 5c). Groups 3 and 4, on the other hand, were the ones with the lowest chances for this disease. It is interesting to note that these two groups (3 and 4) are very distinct in all variables (Figure 4), suggesting that the determinants associated with protection against this disease are distinct in each landscape. Group 3 has the largest extension of continuous forest and lower forest fragmentation; group 4 has a transition area with the Cerrado, which are areas with few forest remnants that have large areas of grain production, especially in southern Mato Grosso and Rondônia. Visceral leishmaniasis is a disease with a predominantly rural history, which, in recent decades, has spanned urban areas and various regions of the country, including eastern Brazilian Legal Amazon ^45^.

Occurrence of malaria and Chagas showed a strong association with group 3, characterized by the predominance of continuous forest (Figures 5d and 5e). Group 5, characterized by mining, also showed a risk for both, although much lower than group 3. The relation of malaria with group 3 is explained by extensive areas of continuous forest since the vector is adapted to the human environment near the forest in flood areas ^46^. As for group 5, in addition to areas of continuous forest, the incidence of malaria may also be associated with mining activities and deforestation, which are predominant in this group, configuring the border municipalities of occupation, such as Itaituba and São Félix do Xingu ^47^.

Municipalities in group 1 failed to statistically differ from group 3 regarding the incidence of Chagas disease. The areas of group 1 with the highest incidence of Chagas mainly lie in Pará. One should note that groups 3 and 1 point to distinct landscapes of this disease, the former is associated with the forest, presence of palm trees (serving as habitats for triatominae) and in which the predominant environmental reservoir of the parasite is located; whereas the latter includes the landscape of environmental degradation associated with the reduction of wild food sources of the triatominae and adaptation to the urban environment ^48^.

Our results are in line with studies that suggest the influence of economic activities on the degradation of local ecosystems and the spread of diseases ^13^ ^,^ ^34^ ^,^ ^49^. This shows that the actions of prevention and control of vector diseases in the Amazon must be aligned with the way of life of the people of the region, observing their relationship with the environment, mediated by the economy and ways of producing ^30^. By assessing environmental and agrarian factors and their impact on disease prevalence, interventions can be more targeted and effective.

The geographical proximity in the groups reflects common dynamics of occupation and land use in neighboring municipalities, indicating the influence of macro-regional processes. Factors such as deforestation, forest degradation, fires, and changes in land use can show a strong spatial dependence, reinforcing the connection between nearby areas. This territorial contiguity is relevant not only to understand the historical patterns of occupation, but also to support territorial planning and, in due course, a more integrated environmental management strategy. By considering joint strategies between municipalities, one can address challenges with shared actions between them, which we believe to be more effective, including climate change adaptation and mitigation actions at local and regional scales.

It should be noted that these analyses have limitations. The environmental indicators fail to include important variables such as the presence of dogs and other hosts relevant to some of these diseases and more specific data from urban areas. Moreover, the use of municipalities as territorial units of analysis constitutes a methodological choice that limits the analysis of results to the municipal scale. When choosing the municipalities of the Amazon as the unit of analysis, results fail to capture intramunicipal heterogeneity due to expected operational issues related to the databases. Regarding the heterogeneity of size between the municipalities and the possible bias related to their size, we emphasize that all indicators were constructed considering the proportion of area in relation to the municipality or the forest area of the municipality (Box 1) to minimize a possible effect related to the area. Even with these limitations, results point to the potential of producing environmental classifications of interest for the epidemiological analysis of the landscape.

## Conclusion

The associations in this study between environmental and land use indicators and the incidence of diseases highlight the importance of assessing the health of ecosystems due to processes of changes in the landscape that affect the provision of ecosystem services, and thus, human well-being and health. Regarding the question that guided this study about the possibility of Amazonian municipalities that have similar environmental and land use characteristics being associated with a specific set of vector diseases, the indicators explored indicate so. They also point to the need to further the analysis and expand the set of indicators, adding other dimensions, such as social and economic ones. Moreover, we highlight the need for interdisciplinary collaboration between public health experts, ecologists, and policymakers to address the complex challenges of the Amazon region. By addressing the interconnected challenges of epidemiology, biodiversity, and occupation of the region, future interventions can contribute to better health outcomes, environmental sustainability, and population well-being.

We also highlight that the conservation of the Amazon rainforest is of great relevance due to its influence on the local, regional, and global climate, the maintenance of biodiversity, the provision of food and water, and the well-being of local and Indigenous communities. Thus, planning and developing strategies for public health interventions should consider conservation status, the modes of production indicated by land uses, and ecosystem restoration processes as integral components of disease prevention actions.
