# Supplementary material for: Epidemiological landscapes in the Amazon: exploring the association between land use, environmental indicators, and vector-borne diseases
Source: Cad Saude Publica. 2025 Jun 9;41(Suppl 1):e00067024. doi: 10.1590/0102-311XEN067024 (PMC12161505; doi:10.1590/0102-311XEN067024)
Supplement: Translation

# Translation: Paisagens epidemiológicas na Amazônia: explorando a associação entre uso da terra, indicadores ambientais e doenças vetoriais

**DOI:** 10.1590/0102-311XPT067024

**Authors:** Ana Claudia Rorato Vitor, Cláudia Torres Codeço, Maria Isabel Sobral Escada

**Affiliations:** 1 Instituto Nacional de Pesquisas Espaciais, São José dos Campos, Brasil.; 2 Programa de Computação Científica, Fundação Oswaldo Cruz, Rio de Janeiro, Brasil.

**Keywords:** Indicadores Ambientais, Ecossistema Amazônico, Vetores de Doenças

## Abstract

Este estudo investiga, por meio do uso de indicadores ambientais, uso da terra e técnicas de análise de agrupamento, características de municípios da Amazônia Legal que podem estar associadas à incidência de doenças vetoriais. Identificamos e descrevemos seis agrupamentos de municípios amazônicos com características ambientais e agrárias similares. Com base em um conjunto de indicadores epidemiológicos, exploramos a incidência de doenças tropicais vetoriais negligenciadas ao longo desses agrupamentos. Os resultados evidenciam uma grande heterogeneidade ambiental dos municípios amazônicos, com perfis diferenciados e bem definidos nos agrupamentos obtidos. Além disso, demonstram a relação de atividades econômicas e degradação ambiental com a propagação de doenças. Essa abordagem permite uma visão abrangente dos desafios ambientais e de saúde enfrentados pela região, contribuindo para a elaboração de estratégias de conservação mais eficazes e adaptadas às necessidades específicas de cada perfil de município.

## Introdução

A Amazônia é reconhecida mundialmente como uma das regiões mais biodiversas e ecologicamente importantes do planeta, desempenhando um papel crucial na regulação do clima regional e global ^1^ ^,^ ^2^. A diversidade cultural, linguística e de assentamentos humanos na região amazônica brasileira é notável, englobando diversas etnias indígenas, comunidades quilombolas e ribeirinhas, além de apresentar uma variedade de núcleos urbanos, que vão desde pequenos núcleos urbanizados, pequenas e médias cidades até centros urbanos industrializados ligados ao Polo Industrial, como Manaus (Amazonas), ou às cadeias de matérias primas (minério e produtos agropecuários) exportadas, como Belém (Pará) ^3^. No entanto, a crescente pressão humana sobre o bioma tem levantado preocupações significativas sobre a sua sustentabilidade a longo prazo. A região está cada vez mais sujeita a impactos resultantes das formas de ocupação que suprimem a floresta, levando ao aumento da temperatura e eventos de secas extremas como o experimentado em 2023 ^4^ ^,^ ^5^, e maior susceptibilidade a incêndios ^6^ ^,^ ^7^ ^,^ ^8^. Estudos sugerem que partes da floresta Amazônica poderão sofrer mudanças estruturais significativas no futuro devido às mudanças climáticas e às intensas conversões de uso e cobertura da terra, acelerando as emissões de carbono e impactando a biodiversidade e a saúde humana, o que também afeta valores socioeconômicos e culturais da região ^9^ ^,^ ^10^.

A atual situação ambiental da Região Amazônica é resultado de múltiplas e contínuas ações humanas que afetam a região historicamente e comprometem sua integridade ambiental ^11^ ^,^ ^12^ ^,^ ^13^. Dentre os principais fatores que afetam a região e levam ao distúrbio e perda florestal, destacam-se a exploração madeireira, o uso do fogo no manejo de áreas com atividades agropecuárias, a grilagem de terras ilegais, a exploração mineral e a expansão da fronteira agropecuária e de projetos de infraestrutura sobre áreas de floresta ^14^ ^,^ ^15^ ^,^ ^16^. A ocorrência e a intensidade desses fatores ameaçam a integridade do bioma e são modulados por uma complexa interação entre fatores sociais, fundiários e econômicos, associados à ocupação histórica dos municípios amazônicos.

Ao longo das últimas décadas, a ocupação da Amazônia ocorreu de maneira heterogênea tanto no tempo quanto no espaço, influenciada por políticas governamentais, interesses econômicos e pressões socioambientais. Enquanto algumas áreas foram colonizadas de forma planejada, por meio da implementação de projetos de assentamento agropecuários e programas de desenvolvimento regional criados pelo governo, outras foram ocupadas de modo espontâneo e desordenado, por meio de processos de migração das populações provenientes do Sul e do Nordeste, principalmente que vieram em busca de terra e de oportunidades econômicas ^17^ ^,^ ^18^. As políticas de ocupação não deram conta de acomodar nos assentamentos rurais todo o contingente populacional que migrou para essa região na década de 1970, sejam eles do Instituto Nacional de Colonização e Reforma Agrária (INCRA) ou de projetos de colonização privada. Essa diversidade na ocupação da paisagem amazônica resultou em uma variedade de padrões de uso da terra, que incluem desde extensas áreas desmatadas para atividades agropecuárias até as áreas de ocupação tradicional, como as terras indígenas, quilombolas, ribeirinhas, além das de proteção integral e de manejo sustentável com uso extrativista.

Diante desse contexto, observa-se que as transformações ambientais e da paisagem ocorridas na Amazônia estão intimamente ligadas à dinâmica da economia agrária regional que foi se estruturando ao longo dos anos ^13^. A heterogeneidade dos padrões de ocupação da terra, assim como a dinâmica da economia agrária, tem implicações significativas nos padrões de degradação ambiental na região. Além disso, a Região Amazônica já sofre os impactos da mudança climática global, evidenciados pelo aumento acentuado das temperaturas, pelas alterações nos padrões de chuva e pelas secas e incêndios intensos que têm afetado o bioma nos últimos anos ^10^ ^,^ ^19^ ^,^ ^20^. Tais mudanças resultam na redução da resiliência da floresta, em mudanças na composição de espécies, mortalidade em massa das árvores e perda irreversível de biodiversidade ^21^ ^,^ ^22^.

Por sua vez, os modos de produção associados aos diferentes sistemas agrários e as transformações na paisagem e na biodiversidade por eles promovidas podem ter impactos diretos na saúde humana ^13^ ^,^ ^23^. Por exemplo, as áreas de manejo sustentável para uso extrativista, que mantém extensas áreas de floresta em pé, desempenham um papel crucial na conservação da biodiversidade e na manutenção dos serviços ecossistêmicos. Por outro lado, a conversão de florestas em terras agrícolas pode levar à perda de habitat de espécies animais, que irão procurar alimento e abrigo em áreas de ocupação humana, aumentando a exposição e o risco de doenças zoonóticas que podem ser transmitidas para os seres humanos. De modo geral, o desmatamento e a fragmentação da paisagem podem desencadear o aumento da abundância de reservatórios de doenças e vetores em contato com as comunidades humanas ^24^ ^,^ ^25^ ^,^ ^26^. Estudos projetam que o risco de doenças transmitidas por vetores na Região Amazônica seja influenciado pelas mudanças climáticas e ambientais ^27^ ^,^ ^28^. Assim, diferentes atividades econômicas podem impactar a paisagem, contribuindo para a emergência e reemergência de doenças ^29^. Leishmanioses, malária, doença de Chagas e dengue são doenças tropicais negligenciadas prevalentes na Região Amazônica, causadas por agentes infecciosos ou parasitas que atingem predominantemente populações vulneráveis ^30^. Elas são indicadores de vulnerabilidade social e ambiental ^13^. Alguns estudos apontam para a associação entre a dinâmica de doenças transmissíveis, como a malária, doença de Chagas, dengue e febre amarela, à perda de biodiversidade e às mudanças na paisagem ^28^ ^,^ ^31^. De acordo com esses estudos, a intensificação da pressão sobre esse bioma gera condições socioambientais críticas que podem desencadear o surgimento de novas doenças infecciosas, provenientes de reservatórios silvestres. Portanto, a saúde ambiental e humana na Amazônia brasileira estão intrinsecamente ligadas, e a economia agrária da região desempenha um papel central nessa interconexão, afetando tanto a saúde das populações locais quanto a saúde do ecossistema amazônico como um todo. Entender a heterogeneidade dos fatores que podem levar a grandes transformações ambientais (dentre elas, a perda de floresta) na Região Amazônica é fundamental para a promoção de um desenvolvimento mais sustentável para toda a região.

Diante desse cenário, a compreensão detalhada dos fatores associados às mudanças ambientais que afetam os municípios da Amazônia brasileira é essencial para trazer informações que possam ajudar na orientação de políticas públicas de conservação e para apoiar tomadas de decisão voltadas à gestão sustentável dos recursos naturais e à saúde humana. Nesse contexto, o presente artigo visa responder a seguinte pergunta científica: municípios amazônicos que apresentam características ambientais e de uso da terra semelhantes podem ser associados a um conjunto específico de doenças vetoriais? A fim de elucidar a questão formulada, nós desenvolvemos uma tipologia com categorias de municípios da Amazônia brasileira que apresentam características ambientais e agrárias em comum, tendo como base um conjunto de indicadores ambientais e de uso da terra. Com essa abordagem, torna-se possível identificar, para cada categoria obtida, os principais fatores econômicos envolvidos nas transformações das paisagens, assim como suas condições ambientais, considerando principalmente os processos de perda florestal e suas implicações na transmissão de doenças vetoriais como malária, doença de Chagas, leishmanioses e dengue. Para alcançar esse objetivo, foram utilizados dados e informações disponíveis sobre uma variedade de indicadores ambientais, de uso da terra e epidemiológicos, incluindo desmatamento, degradação florestal, áreas queimadas por incêndios e mineração ^32^.

## Método

### Área de estudo

A área de estudo contemplou todos os municípios inteiramente dentro da região da Amazônia Legal brasileira. A Amazônia Legal brasileira é uma região político-administrativa que abrange cerca de 5 milhões de km^2^ e corresponde a aproximadamente 58,9% do território brasileiro. Essa região compreende os estados do Acre, Amapá, Amazonas, Pará, Rondônia, Roraima, Mato Grosso, Tocantins e parte do Maranhão. Em relação aos biomas brasileiros, a Amazônia Legal abriga todo o bioma Amazônico (composto principalmente por florestas tropicais), 20% do bioma Cerrado (composto predominantemente por vegetação de savana) e cerca de 40% do bioma Pantanal (planície de inundação), no Estado de Mato Grosso. A floresta tropical amazônica desempenha papel central na manutenção do ciclo hidrológico regional, contribuindo por meio da reciclagem e transporte de umidade dentro e fora da região ^33^. O desmatamento resultante de múltiplas transições de uso e cobertura da terra representa uma ameaça imediata às florestas, sendo o desmatamento para pastagens a transição mais frequente e de alto impacto na Amazônia brasileira ^34^ ^,^ ^35^. Outras transições de alto impacto, que ocorrem com menor frequência ou com menor extensão de área, porém com grande importância, são as conversões para a agricultura ^34^ e mineração ^36^. De forma geral, a intensificação ou diminuição da degradação do bioma amazônico respondem às mudanças nas políticas ambientais, como a fiscalização do desmatamento e das atividades associadas ao mercado ilegal de terras, o incentivo para atividades econômicas, a construção de infraestruturas que possibilitam o escoamento da produção agropecuária (como estradas, portos e hidrelétricas) e a oscilação dos preços das commodities. Segundo o Projeto de Monitoramento do Desmatamento na Amazônica Brasileira por Satélite (PRODES), o valor estimado do desmatamento acumulado para o ano de 2023 foi de 9.001km^2^, representando uma redução de 22,37% em relação à taxa de desmatamento consolidada pelo PRODES 2022, que foi de 11.594km^2^ para os nove estados da Amazônia Legal, mas ainda se mantendo em um alto patamar. As grandes e rápidas transformações da paisagem florestal podem levar à desequilíbrios nesse ecossistema e, por consequência, provocar mudanças nos ciclos de transmissão de doenças vetoriais, devido à perda de habitat e à proximidade da população humana aos remanescentes florestais, vetores e reservatórios, levando à uma maior exposição à doenças vetoriais.

### Indicadores ambientais e epidemiológicos

Nós utilizamos um conjunto de indicadores ambientais para caracterizar o perfil ambiental dos municípios amazônicos observando as seguintes dimensões: mudanças ambientais, uso e cobertura da terra (LULC − *land use and land cover*) e anomalias climáticas. Compondo a dimensão de mudança ambiental temos os indicadores de desmatamento, degradação florestal, incêndios, mineração e fragmentação florestal. A dimensão LULC é representada por indicadores de agricultura, pastagens, área urbanizada e vegetação secundária. Indicadores de anomalias de precipitação e temperatura representam a dimensão de anomalias climáticas. Os indicadores foram calculados para todos os municípios da Amazônia Legal e foram construídos a partir de bases de dados abertas e consolidadas. Nós também exploramos a associação dos agrupamentos de municípios com a predominância de doenças tropicais negligenciadas vetoriais. Para isso, utilizamos um conjunto de indicadores epidemiológicos de incidência de leishmaniose cutânea (tegumentar) e visceral, malária, doença de Chagas e dengue para o período de 2015-2019, a partir das bases de dados do DATASUS (Departamento de Informática do Sistema Único de Saúde). A taxa de incidência é uma medida da frequência da doença em populações dinâmicas, e foi estimada dividindo o número de casos pela população no meio do período e multiplicando por cinco (quantidade de anos de observação). Todos os indicadores ambientais e epidemiológicos utilizados neste estudo são descritos detalhadamente em Rorato et al. ^32^ e estão disponíveis para acesso em: https://zenodo.org/records/7098053. Os indicadores ambientais, de uso da terra e epidemiológicos estão resumidos no Quadro 1.

**Quadro 1:** Indicadores ambientais, de uso da terra e epidemiológicos.

DIMENSÃO	INDICADOR	DESCRIÇÃO	UNIDADE	PERÍODO	ID
Mudança ambiental	Desmatamento	a área total de desmatamento no município entre 2010 a 2016 é dividida pela sua área florestal original	km^2^/km^2^	2010-2016	desmat
Mudança ambiental	Degradação florestal	a área total de degradação florestal no município entre 2007 a 2017 é dividida pela sua área florestal original	km^2^/km^2^	2007-2017	degrad
Mudança ambiental	Fogo	proporção da área do município que sofreu pelo menos um incêndio entre 2012 e 2017	km^2^/km^2^	2012-2017	fogo
Mudança ambiental	Mineração	proporção da área do município utilizada para mineração (industrial e artesanal) em 2017	km^2^/km^2^	2017	mine
Mudança ambiental	Fragmentação	proporção da área do município classificada como núcleo de vegetação natural em 2017	km^2^/km^2^	2017	nucleo
Mudança ambiental	Fragmentação	razão entre a borda total (perímetro) das manchas de vegetação natural do município e a raiz quadrada da área total dessas manchas, em 2017	m/sqrt(m^2^)	2017	borda
LULC	Vegetação secundária	proporção da área do município coberta por vegetação secundária em 2017	km^2^/km^2^	2017	veg_sec
LULC	Pastagem	proporção da área do município classificada como pastagem em 2017	km^2^/km^2^	2017	pasto
LULC	Agricultura	proporção da área do município classificada como agricultura em 2017	km^2^/km^2^	2017	agric
LULC	Área urbana	proporção da área do município classificada como urbana em 2017	km^2^/km^2^	2017	urb
Anomalias climáticas	Precipitação	média da área de anomalia de precipitação positiva observada durante as estações secas de 2007-2017, dividida pela área total	m^2^/m^2^	2007-2017	precp+
Anomalias climáticas	Precipitação	média da área de anomalia de precipitação negativa observada durante as estações secas de 2007-2017, dividida pela área total	m^2^/m^2^	2007-2017	precp-
Anomalias climáticas	Temperatura	média da área de anomalia de temperatura positiva observada durante as estações frias de 2007-2017, dividida pela área total	m^2^/m^2^	2007-2017	temp+
Epidemiológico	Doenças transmitidas por vetores	incidência de doença de Chagas, leishmaniose cutânea, leishmaniose visceral, dengue, malária	Taxa de incidência	2015-2019	inc_

ID: identificador; LULC: uso e cobertura da terra (*land use and land cover*, acrônimo em inglês).

### Análise de agrupamentos

Com o propósito de identificar grupos de municípios na Amazônia que enfrentam mudanças ambientais similares, conduzimos uma análise de agrupamentos utilizando todas as variáveis ambientais acima descritas. Essa técnica não supervisionada visa dividir um conjunto de dados em grupos similares, comumente chamados de *clusters*, visando maximizar a homogeneidade *intra-cluster* e a heterogeneidade *inter-cluster* ^37^. Nós utilizamos o método de agrupamento *k-means*, uma técnica não hierárquica amplamente empregada devido à sua simplicidade e eficácia, em que o usuário determina *a priori* o número de *clusters*, *k*. Nesse método, são selecionados *k* objetos (municípios) como centros iniciais, e os demais são alocados com base na similaridade, garantindo a menor distância entre cada objeto e o centro do *cluster*. O centro de cada *cluster*, conhecido como centro de gravidade, é calculado como a média dos objetos do *cluster*. Neste estudo, a distância euclidiana quadrada foi utilizada como medida de similaridade. Esse método permitiu agrupar municípios com características ambientais semelhantes com base nos valores dos indicadores ambientais e agrários.

Antes da análise, aplicamos uma transformação logarítmica nos indicadores para melhorar a normalidade de distribuição das variáveis. Em seguida, padronizamos os indicadores logarítmicos usando o método *z-score*, visando melhorar a precisão do algoritmo *k-means*, o qual se baseou na distância euclidiana das observações ^38^ ^,^ ^39^. O número ideal de *clusters* foi determinado utilizando o método de Elbow, que consiste em plotar a variação explicada em função do número de grupos. A escolha do número de grupos é feita observando o gráfico e escolhendo o número de grupos de acordo com o cotovelo da curva, ou seja, o número de grupos em que a curva plotada começa a se assemelhar a uma linha reta. Todas as análises e visualizações foram conduzidas utilizando os pacotes *stats* e *factoextra* no software R (http://www.r-project.org) e o aplicativo Quantum Gis 3.0 (https://qgis.org/en/site/).

### Modelagem da distribuição de doenças transmitidas por vetores nos agrupamentos ambientais

Modelos lineares generalizados foram utilizados para identificar associações entre a incidência das doenças de transmissão vetorial e os agrupamentos de municípios gerados pela classificação ambiental. Foram testados modelos com distribuição de Poisson, binomial negativa e binomial negativa com inflação de zeros, no ambiente R, utilizando as bibliotecas *MASS* e *pscl*. Todos tiveram como desfecho o total de casos no município (período de 2015 a 2019), e como *offset* o logaritmo da população estimada em 2017, com a classificação do agrupamento ambiental sendo a única variável explicativa. Para a dengue e as leishmanioses, o modelo binomial negativo teve a melhor qualidade de ajuste, medido pelo critério de informação de Akaike (AIC). Para malária e Chagas, esse não convergiu, e foi necessário recorrer ao modelo inflacionado de zeros.

## Resultados e discussão

### Classificação ambiental e de uso da terra dos municípios

Com base no método de Elbow, nós encontramos 6 grupos (*k* = 6) em nossa análise. Cada grupo é composto por um conjunto de municípios com perfil ambiental similar, cuja distribuição espacial é apresentada na Figura 1. A Figura 2 apresenta a distribuição dos grupos de municípios no espaço multivariado, cujos eixos são as três primeiras componentes principais. Em relação a separação dos grupos, é possível observar que os grupos 1, 3 e 6 são bem separados em relação ao eixo 1, enquanto o *cluster* 6 se destaca em relação ao eixo 2 (Figura 2a). O eixo 2 também separa o grupo 5 de todos os demais. Já os grupos 2 e 4 são melhor evidenciados em relação ao eixo 3 (Figura 2b). Nossos resultados evidenciam uma grande heterogeneidade ambiental dos municípios Amazônicos (Figuras 3 e 4), com perfis bem definidos em cada agrupamento de maneira geral. As características ambientais de cada grupo são sumarizadas nas Figuras 3 e 4 e são discutidas a seguir.

**Figura 1:** Classificação ambiental dos municípios da Amazônia Legal brasileira em 2017, obtida pela análise de agrupamentos (*k-means*).

**Figura 2:** Distribuição dos municípios da Amazônia Legal brasileira no espaço multivariado gerado pelas variáveis ambientais do estudo.

Nota: as cores indicam os grupos obtidos a partir do método de agrupamento *k*-*means*.

**Figura 3:** Gráficos de radar indicando a distribuição das variáveis ambientais em cada agrupamento de municípios da Amazônia Legal brasileira.

Nota: os valores indicam a média de cada indicador para cada grupo. Para melhorar a visualização dos resultados neste gráfico, aplicamos transformação logarítmica seguida de escalonamento entre 0 e 1 (utilizando o método mínimo-máximo) nas variáveis originais.

**Figura 4:** *Boxplots* dos indicadores ambientais ao longo dos agrupamentos de municípios da Amazônia Legal brasileira.

Nota: os eixos aumentam quadraticamente para melhorar a visibilidade das diferenças. Os limites superior e inferior correspondem ao primeiro e terceiro quartis, e a linha dentro da caixa representa a mediana. Os dados além do final dos limites são *outliers* e plotados como pontos.

O agrupamento 1 é composto por 169 municípios, sendo o segundo maior agrupamento. Os municípios desse agrupamento estão em sua maioria localizados nos Estados do Pará, Mato Grosso e Maranhão, assim como alguns municípios isolados no interior dos estados do Amapá, Roraima, Amazonas, Rondônia, Tocantins e Acre (Figura 1). Esse agrupamento se destaca por apresentar municípios com as maiores médias de desmatamento, degradação florestal e efeito de borda florestal entre todos os grupos. Fazem parte desse grupo municípios como Santarém (Oeste do Pará), Novo Progresso e Jacareacanga (Sudoeste do Pará) e Colniza (Noroeste do Mato Grosso), que apresentam grandes áreas florestais, muitas vezes concentradas em unidades de conservação ou Terras Indígenas. Entretanto, parte desses municípios, como Novo Progresso e Colniza, também figuram entre os 10 municípios que mais desmataram no período considerado até 2023 ^40^. O grupo também apresenta a segunda maior média em áreas queimadas (Figuras 3 e 4). No caso do desmatamento, práticas como agricultura mecanizada em larga escala e pecuária extensiva são impulsionadas pelo mercado de terras e pela conversão de áreas florestais para atender à demanda por *commodities*.

O agrupamento 2 é formado por 201 municípios, sendo o maior agrupamento de todos. Os municípios desse agrupamento estão concentrados principalmente nos estados do Maranhão, Tocantins, Pará, Acre e Rondônia (Figura 1). São mais numerosos no Estado do Pará, concentrando-se na região nordeste (Zona da Bragantina) e fazendo vizinhança com os municípios do Estado do Maranhão, que também estão incluídos nesse grupo. São municípios em que grande parte dos sistemas agrícolas são realizados em sistemas de *pousio*. Nesses sistemas, a fertilidade do solo é recuperada por meio do pousio, no qual após a colheita há o abandono da terra e crescimento da vegetação secundária, em intervalos de tempos que podem variar de região para região ^5^ ^,^ ^41^. Fazem parte desse agrupamento municípios ribeirinhos dos estados do Amazonas e do Pará, em que o sistema de pousio também é prática comum. Os municípios desse agrupamento possuem as maiores médias de área de vegetação secundária, urbanização e anomalia de temperatura positiva (Figuras 3 e 4). São municípios que apresentaram pouco desmatamento no período analisado, seja porque apresentam grandes áreas de floresta primária ou porque, ao contrário, apresentam pequenos remanescentes de floresta primária, mas uma proporção grande de vegetação secundária em sua substituição.

O agrupamento 3 é formado por 111 municípios localizados principalmente nos estados do Amazonas, Roraima, Acre, Pará e Amapá (Figura 1). Os municípios desse grupo se localizam na região ocidental da Amazônia, com maior proporção de área de floresta, destacando-se por apresentarem as maiores médias da porcentagem de área de núcleo florestal em comparação aos demais grupos. Em contrapartida, possuem os menores valores para fogo, pastagens, agricultura, urbanização e anomalia de precipitação negativa (Figuras 3 e 4), indicando que são municípios que apresentam menores distúrbios ambientais e floresta mais íntegra.

O agrupamento 4 é composto por 90 municípios localizados principalmente em áreas de uso agropecuário consolidado, concentrando-se principalmente nos estados do Mato Grosso e Rondônia (Figura 1). Esses municípios são caracterizados ambientalmente pelas maiores médias de áreas sob efeito de borda florestal (empatando com o grupo 1), agricultura e anomalias de precipitação positiva. No Estado do Mato Grosso, os municípios indicados podem apresentar grandes proporções de áreas de fitofisionomia não florestal ou de floresta estacional. Além disso, esse agrupamento é o que apresenta a segunda maior área de pastagens, degradação florestal e de anomalias de precipitação negativa (Figuras 3 e 4).

O agrupamento 5 é composto por 43 municípios, localizados principalmente nos estados do Pará, Rondônia, Mato Grosso e Amapá (Figura 1). Esse grupo se destaca por apresentar municípios com maior a média de porcentagem de área de mineração e a segunda maior média de porcentagem de desmatamento em relação aos demais grupos. Destacam-se também a predominância de áreas sob efeito de borda e de degradação florestal (Figuras 3 e 4). Nesse agrupamento, encontram-se municípios como Itaituba e Jacareacanga (oeste do Pará) e São Felix do Xingu e Ourilândia do Norte (leste do Pará), onde a atividade de mineração ilegal tem se intensificado muito nos últimos anos, inclusive no interior de Terras Indígenas como a Kayapó e Munduruku, com significativos impactos sobre as populações locais ^42^. Esses municípios apresentam grandes áreas florestais devido à presença de Unidades de Conservação e Terras Indígenas, e nas áreas desmatadas, a principal cobertura é a pastagem, ainda que o agrupamento não apresente a maior média de áreas de pastagem dos agrupamentos.

O agrupamento 6 é o terceiro maior agrupamento, composto por 159 municípios localizados principalmente nos estados do Tocantins, sul do Maranhão e do Mato Grosso (Figura 1). São municípios pequenos, que se encontram em região de transição com o bioma cerrado e que podem apresentar grandes manchas de vegetação com fitofisionomias do cerrado. Esse agrupamento se destaca por apresentar as maiores médias de anomalia de precipitação negativa, de áreas de pastagens e área queimada, e também por ter a segunda maior média de área de agricultura anual, vegetação secundária e anomalias de temperatura positiva (Figuras 3 e 4).

### Associação das doenças de transmissão vetorial com a classificação ambiental

Nessa etapa, nós exploramos a suposição de que a classificação ambiental e de uso da terra produzida pelo estudo se relaciona com a distribuição da incidência de algumas doenças vetoriais na região. Para as cinco doenças estudadas, a inclusão da variável “grupo” resultou em melhor ajuste, medido por análise de *Deviance*. A Figura 5 e a Tabela 1 apresentam os *odds ratios* (OR) de ocorrência dos agravos por grupo ambiental, calculados tendo o grupo “3” como referência. Esse grupo é o conjunto de municípios com maior preservação do bioma.

**Figura 5:** Associação entre a incidência de doenças de transmissão vetorial e os agrupamentos gerados pela classificação ambiental dos municípios da Amazônia Legal brasileira.

Nota: a barra indica a razão de chance de ocorrência do caso no agrupamento em comparação com o agrupamento 3 (maior integridade ecológica). A descrição dos grupos está nas Figuras 3 e 4 e a localização, na Figura 1

**Tabela 1:** Resultado do ajuste dos modelos lineares generalizados.

Doença/Grupo	OR	IC95%
Dengue		
1	1,50	1,12-2,01
2	0,69	0,52-0,91
3	1,00	1,00-1,00
4	1,72	1,23-2,42
5	1,29	0,85-2,02
6	1,50	1,12-2,02
Leishmaniose tegumentar		
1	0,86	0,66-1,13
2	0,35	0,27-0,45
3	1,00	1,00-1,00
4	0,77	0,57-1,06
5	0,75	0,51-1,12
6	0,36	0,27-0,47
Leishmaniose visceral		
1	3,08	1,88-4,98
2	3,82	2,37-6,08
3	1,00	1,00-1,00
4	0,98	0,55-1,79
5	2,44	1,25-5,03
6	3,19	1,93-5,21
Malária		
1	0,07	0,04-0,13
2	0,02	0,01-0,03
3	1,00	1,00-1,00
4	0,02	0,01-0,05
5	0,20	0,09-0,42
6	0,00	0,00-0,01
Doença de Chagas		
1	0,33	0,11-1,04
2	0,18	0,08-0,39
3	1,00	1,00-1,00
4	0,01	0,00-0,07
5	0,27	0,09-0,85
6	0	NO

IC95%: intervalo de 95% de confiança; NO: não observado; OR: *odds ratio*.

A dengue e a leishmaniose tegumentar apresentaram alta incidência em todos os agrupamentos ambientais (Figura 6). Contudo, a dengue tem as maiores incidências nos grupos 1, 4 e 6 (Figura 5a), que se concentram a Sul e Sudeste da região amazônica, sendo áreas de grande transformação ambiental e que compartilham características como a alta porcentagem de área queimada no período, fragmentação das florestas, forte presença da agricultura, pastagem, maior proporção de vegetação secundária e maior ocorrência de anomalias de precipitação negativa dentre os grupos. Por outro lado, a dengue tem a menor incidência no grupo 2, seguida do grupo 3. Esses grupos compartilham menor área de queimadas, menor degradação e fragmentação florestal, além de menor extensão de área agrícola.

**Figura 6:** *Boxplots* dos indicadores epidemiológicos ao longo dos agrupamentos de municípios da Amazônia Legal brasileira.

Nota: os eixos aumentam quadraticamente para melhorar a visibilidade das diferenças. Os limites superior e inferior correspondem ao primeiro e terceiro quartis, e a linha dentro da caixa representa a mediana. Os dados além do final dos limites são *outliers* e plotados como pontos.

A leishmaniose tegumentar teve incidência estatisticamente similar nos grupos 1, 3, 4 e 5, que representam 53% de todos os municípios. São municípios que apresentam grande proporção de remanescentes de florestas, com variados graus de desmatamento e fragmentação florestal. Apenas os grupos 2 e 6 apresentaram menor chance dessa doença (Figura 5b). Esses dois grupos compartilham algumas características: menores áreas de núcleos florestais, maiores áreas com vegetação secundária e maior ocorrência de anomalia de temperatura positiva. Historicamente, a leishmaniose cutânea é associada a municípios amazônicos com grande proporção de área florestal, especialmente em comunidades extrativistas. Porém, o avanço da ocupação humana e da urbanização na região levou a leishmaniose cutânea para outras paisagens ^43^, o que explica a sua ampla distribuição pela região, mas ainda assim apresenta uma relação com a presença da floresta. Novos focos dessa doença são associados a áreas com desmatamento recente para implantação de agricultura ^44^, o que é coerente com os resultados encontrados.

A leishmaniose visceral teve maior incidência nos municípios dos grupos 1, 2, 5 e 6, num padrão bem diferente da leishmaniose tegumentar (Figura 5c). Já os grupos 3 e 4 foram os que apresentaram as menores chances para essa doença. Interessante notar que esses dois grupos (3 e 4) são muito distintos em todas as variáveis (Figura 4), sugerindo que os determinantes associados à proteção a essa doença são distintos em cada paisagem. No grupo 3, tem-se a maior extensão de floresta contínua e menor fragmentação florestal; no grupo 4, tem-se área de transição com o cerrado e são áreas com poucos remanescentes florestais e que apresentam grandes áreas de produção de grãos, principalmente no sul dos estados do Mato Grosso e de Rondônia. A leishmaniose visceral é uma doença de histórico predominantemente rural, que nas últimas décadas apresentou expansão para áreas urbanas e para várias partes do país, incluindo a região oriental da Amazônia Legal brasileira ^45^.

Ocorrência de malária e Chagas apresentou forte associação com o grupo 3, caracterizado pela predominância de floresta contínua (Figuras 5d e 5e). O grupo 5, caracterizado pela mineração, também apresentou risco para ambas, embora bem mais baixo que o grupo 3. A relação da malária com o grupo 3 é explicada pela presença de áreas extensas de floresta contínua, uma vez que o vetor é adaptado ao ambiente humano próximo da mata em áreas de inundação ^46^. Quanto ao grupo 5, além das áreas de floresta contínua, a incidência de malária também pode estar associada às atividades de mineração e ao desmatamento, que são predominantes nesse grupo, configurando os municípios de fronteira de ocupação, como Itaituba e São Félix do Xingu ^47^.

Municípios do grupo 1 não diferiram estatisticamente do grupo 3 quanto à incidência de Chagas. Essas áreas do grupo 1 de maior incidência de Chagas estão concentradas no Pará. Interessante notar que os grupos 3 e 1 apontam para paisagens distintas dessa doença, a primeira associada à floresta, com presença de palmeiras que são habitats do triatomíneo, e onde está o reservatório ambiental predominante do parasita; o segundo, com a paisagem de degradação ambiental associada à redução das fontes de alimentação silvestres do triatomíneo e à adaptação ao meio urbano ^48^.

Nossos resultados estão alinhados com estudos que sugerem a influência de atividades econômicas na degradação dos ecossistemas locais e na propagação de doenças ^13^ ^,^ ^34^ ^,^ ^49^. Isso evidencia que as ações de prevenção e controle das doenças vetoriais na Amazônia precisam estar alinhadas com o modo de viver das pessoas da região, observando sua relação com o ambiente, mediada pela economia e pelos modos de produzir ^30^. Ao avaliar fatores ambientais e agrários e seu impacto na prevalência das doenças, as intervenções podem ser mais direcionadas e eficazes.

A proximidade geográfica observada nos agrupamentos reflete dinâmicas comuns de ocupação e uso da terra entre municípios vizinhos, indicando a influência de processos macrorregionais. Fatores como desmatamento, degradação florestal, fogo e mudanças no uso da terra podem apresentar forte dependência espacial, reforçando a conexão entre áreas próximas. Essa contiguidade territorial é relevante, não apenas para compreender os padrões históricos de ocupação, mas também para dar suporte ao planejamento territorial e, oportunamente, à uma estratégia de gestão ambiental mais integrada. Ao considerar estratégias conjuntas entre municípios, é possível enfrentar desafios com ações compartilhadas entre eles, o que acreditamos ser mais eficaz, incluindo ações de adaptação e mitigação às mudanças climáticas em escalas locais e regionais.

Cabe ressaltar que existem limitações nas análises apresentadas, já que os indicadores ambientais não incluem variáveis importantes como a presença de cães e outros hospedeiros relevantes para alguns desses agravos, e também não estão incluídos dados mais específicos de áreas urbanas. Além disso, reconhecemos que o uso dos municípios como unidades territoriais de análise constitui uma escolha metodológica que limita a análise dos resultados à escala municipal. Ao escolher os municípios da Amazônia como unidade de análise devido a questões operacionais relacionadas com as bases de dados, observa-se que a heterogeneidade intramunicipal não é capturada nos resultados, como era de se esperar. Em relação a heterogeneidade de tamanho entre os municípios e o possível viés relacionado ao tamanho dos mesmos, destacamos que todos os indicadores foram construídos levando em conta a proporção de área em relação ao município ou à área de floresta do município (Quadro 1), objetivando minimizar um possível efeito relativo à área. Mesmo com essas limitações, os resultados apontam para o potencial de produção de classificações ambientais de interesse para a análise epidemiológica da paisagem.

## Conclusão

As associações observadas neste estudo entre indicadores ambientais e de uso da terra com a incidência de doenças realça a importância de se avaliar a saúde dos ecossistemas, resultante de processos de mudanças na paisagem, que afetam a provisão dos serviços ecossistêmicos e consequentemente o bem-estar e a saúde humana. Em relação à pergunta que norteou este trabalho, sobre a possibilidade dos municípios amazônicos que apresentam características ambientais e de uso da terra semelhantes poderem ser associados a um conjunto específico de doenças vetoriais, os indicadores explorados apontam que sim. Apontam também para a necessidade de aprofundamento das análises e para a ampliação do conjunto de indicadores, acrescentando outras dimensões como a social e a econômica. Em adição, destacamos a necessidade de colaboração interdisciplinar entre especialistas em saúde pública, ecologistas e formuladores de políticas para enfrentar os complexos desafios da Região Amazônica. Ao abordar os desafios interligados da epidemiologia, da biodiversidade e da ocupação da região, as intervenções futuras podem contribuir para melhores resultados de saúde, sustentabilidade ambiental e bem-estar da população.

Destacamos ainda que a conservação da floresta amazônica é de grande relevância devido à sua influência no clima local, regional e global, na manutenção da biodiversidade, provisão de alimentos e água e no bem-estar das comunidades locais e indígenas. Assim, no planejamento e no desenvolvimento de estratégias de intervenções em saúde pública, o estado de conservação, os modos de produção indicados pelos usos da terra e os processos de restauração dos ecossistemas devem ser considerados como componentes integrantes das ações de prevenção a doenças.
